# Molecular mechanisms of m6A modifications regulating tumor radioresistance

**DOI:** 10.1186/s10020-025-01121-9

**Published:** 2025-02-19

**Authors:** Ruolin Shen, Zhenyang Jiang, Huanhuan Wang, Zhuangzhuang Zheng, Xin Jiang

**Affiliations:** 1 Jilin Provincial Key Laboratory of Radiation Oncology and Therapy, The First Hospital of Jilin University, Changchun, China; 2 Department of Radiation Oncology, The First Hospital of Jilin University, 71 Xinmin Street, Changchun, 130021 China; 3 NHC Key Laboratory of Radiobiology, School of Public Health, Jilin University, Changchun, China

**Keywords:** Cancer, m6A methyltransferase, m6A modification, radioresistance

## Abstract

Radiotherapy is one of the most effective treatments for malignant tumors. Radioresistance is a major factor that contributes to radiotherapy failure and poor prognosis. Recent studies have elucidated the pivotal role of aberrant N6-methyladenosine (m6A) modification, the predominant internal mRNA modification in eukaryotic cells, influences cancer progression by disrupting gene expression and other critical cellular processes. Furthermore, aberrant m6A methylation provides a substrate for tumor therapy; however, whether it regulates tumor radioresistance remains unclear. Methylated transferase (writer), demethylated transferase (eraser), and methylated recognition protein (reader) are the three essential proteins that regulate m6A modification via different mechanisms in different tumors. This review summarizes the latest research advances in m6A methylation and aims to provide novel perspectives on the advancement of regimens to overcome radioresistance and tumor invasion.

## Background

Radiotherapy is one of the most essential forms of treatment for malignant tumors and is used in more than 70% of patients with cancer (Galluzzi et al. 2023). With an accelerating technological evolution, the efficacy of radiotherapy continues to improve; however, some tumors still resist radiotherapy, leading to disease recurrence (Kim et al. 2015). Tumor radiosensitivity is a central factor governing the outcomes of radiotherapy. With the increasing morbidity associated with malignant tumors, it is crucial to develop more effective strategies to improve radiosensitivity.

Radiosensitivity describes the variable reactions of cells, tissues, organs, and individuals exposed to ionizing irradiation (Zhou et al. 2023). Radiosensitivity is correlated with the organ origin and pathological type of tumors and also depends on numerous factors including age, genetic predisposition, and lifestyle patterns (Nachef et al. 2021). Furthermore, radiosensitivity is modulated by multiple epigenetic modifications and signaling pathways, including DNA methylation, non-coding RNA (ncRNA) regulation, and posttranslational modifications (Jurkovicova et al. 2022) Several recent studies have indicated that m6A modification affects radiosensitivity. N6-adenosine methylation (m6A) represents the most prevalent and enriched internal co-transcriptional modification within the mRNAs of higher eukaryotes, accounting for roughly 0.1–0.4% of all adenosines (Jiang et al. 2021; Meyer et al. 2012). m6A modifications participate in RNA metabolism by regulating alternative splicing, nuclear export, translation, degradation, and ncRNA processing (Sun et al. 2019; Liu et al. 2018; Zhang et al. 2020).

**Fig. 1:**
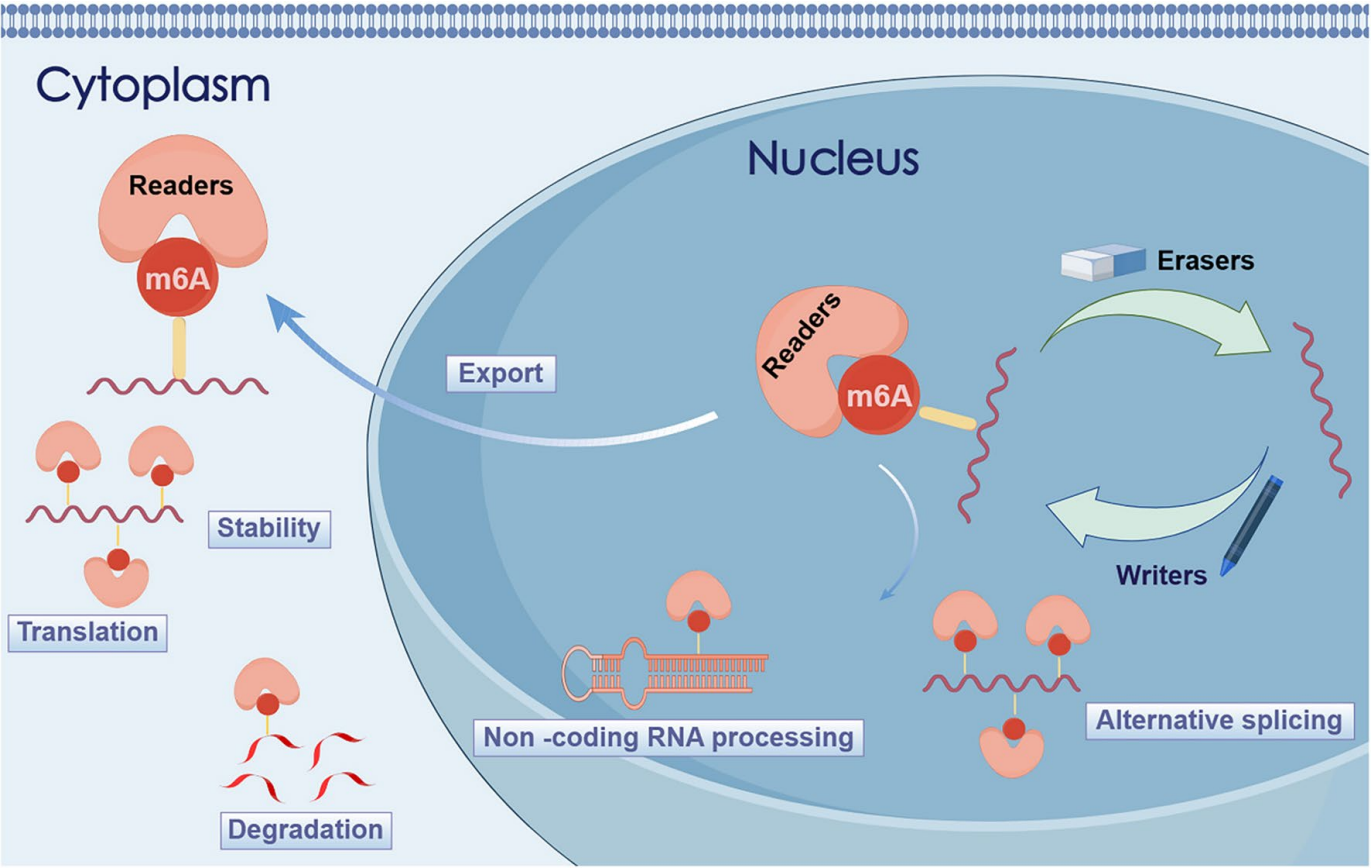
Introduction to the functions of m6A regulatory factors (By Figdraw.) Methylated transferase (writer), demethylated transferase (eraser), and methylated recognition protein (reader) regulate alternative splicing, nuclear export, translation, stability, degradation, and ncRNA processing by adding, removing or recognizing sites modified by m6A, leading to RNA metabolism

Three major classes of enzymes are involved in m6A modification: methylated transferases (writers), demethylated transferases (erasers), and methylated recognition proteins (readers) (Xu et al. 2022a). These enzymes act as regulators of m6A RNA methylation, by modulating biological processes through the addition, removal, or recognition of sites modified by m6A (Fig. 1) (Li et al. 2021). The abnormal level of m6A mainly depends on the “writers” and “erasers”, whereas the fate of the target RNA after modification is mostly directed by the reader and is independent of transcript sequence or function. The m6A reader is divided into three categories, with Class I including the YTH domain, which is the main reader. YTHDC1 mediates RNA splicing, promotes ribosome assembly, and interacts with initiation factors to promote the translation of m6A methylated mRNA (Bao et al. 2023). YTHDC2, located on human chromosome 5, may play a role in regulating mRNA translation and stability by recognizing m6A modification (Wang et al. 2023a). YTHDF2 selectively recruits mRNA decay sites to accelerate the decay of m6A methylated mRNA. YTHDF2 and YTHDF1 exert antagonistic functions to regulate mRNA stability (Ma et al. 2023). YTHDF3 and YTHDF1 work synergistically to promote RNA translation. The interaction between YTHDF3 and YTHDF2 promotes mRNA decay (Zaccara and Jaffrey 2020). YTHDC1 is primarily located in the nucleus and is required for pre-mRNA splicing, RNA export, and mRNA destabilization. YTHDC2 interacts with RNA helicase, exhibiting 3′-5′ RNA helicase activity, improving the efficiency of translating targets (Hsu et al. 2017). Class II m6A readers include three types of heterogeneous nuclear ribonucleoproteins (HNRNPs), HNRNPC, HNRNPG, and HNRNPA2B1. HNRNPC is an abundant nuclear RNA-binding protein responsible for pre-mRNA processing (Liu et al. 2015). HNRNPG uses Arg Gly Gly (RGG) motifs to co-transcriptionally interact with both RNA polymerase II and m6A-modified nascent pre-mRNA to modulate RNAPII occupancy and alternative splicing (Zhou et al. 2019). HNRNPA2B1 binds to m(6)A marks in a subset of primary miRNA transcripts, interacts with the microRNA microprocessor complex protein DGCR8, and promotes primary miRNA processing (Alarcón et al. 2015). Class III readers include insulin-like growth factor 2 mRNA-binding proteins (IGF2BPs), a conserved family of single-stranded RNA-binding proteins, which prevent the decay of m6A-modified mRNAs. IGF2BPs can facilitate the stability and the translation efficiency in an m6A-dependent fashion by recognizing consistent GG (m6A) C sequences and binding to target transcripts (Huang et al. 2018).

m6A modification performs pivotal roles in numerous biological processes including malignant tumor occurrence, development, proliferation, metastasis, and drug resistance (Fang et al. 2022). Previous studies have reported the significant impact of m6A modifications on radiosensitivity. Certainly, poly (ADP-ribose) polymerase 1 can modulate associated RNA m6A methylation and the functionality of target genes to control radiosensitivity (Sun et al. 2023) in cancer treatment. These findings suggest that the radiosensitivity of tumors may be increased by modulating m6A modifications.DNA damage repair, cell cycle arrest, cancer stem cell generation, alterations in the tumor microenvironment (TME) and hypoxia are the main contributors to tumor radiosensitivity (Mayerhofer et al. 2023). In this article, we mainly review the modulation of radiosensitivity by m6A modifications in these five areas. This review aimed to update our understanding of m6A modifications and provide new insights for developing interventions to increase the radiosensitivity of tumors.

### Regulation of DNA damage repair by m6A modifications

Irradiation attacks tumor cells by directly damaging DNA and indirectly stimulating the generation of reactive oxygen species (ROS). DNA double-stranded breaks (DSBs), representing one of the most significant patterns of DNA damage and are fatal because of their genomic instability and chromosomal rearrangements (Liu et al. 2023). In eukaryotic cells, there are two routes for DSB repair: non-homologous end-joining (NHEJ) and homologous recombination (HR) (Fu et al. 2019). The NHEJ pathway repairs DSBs throughout the entire cell cycle, accounting for 80–90% of all damage (Chang et al. 2017). However, HR is generally confined to repairing DSBs in the S phase and G2/M phases (Vitale et al. 2017). The survival and death of tumor cells are determined by the efficiency and accuracy of DSB repair following radiation. Therefore, inhibiting DNA damage repair by targeting key molecules participating in DSB repair will significantly contribute to the downgrading of tumor radioresistance (Table 1).

**Table 1:** Role of m6A methylation modulators in regulating radiosensitivity mediated by the DNA damage response

m6A regulator	Type	Cancer types	Roles	Target factor	Mechanism	References
METTL3	Writer	GBM	Oncogene	SOX2	Priority activation of DNA damage checkpoints and control of homologous recombination in GSC	Xie et al. (2022)
METTL3 YTHDF2	Writer Reader	GBM	Oncogene	LINC00839	Prioritize the activation of checkpoint Chk1/2 and ATM, and promote the expression of HR related and MHEJ related proteins.	Yin et al. (2023)
METTL3	Writer	NSCLC	Oncogene	H2AX	Promote H2AX expression, thereby enhancing DNA damage repair and cell survival	Xu et al. (2023)
METTL3 YTHDF2	Writer Reader	CC	Oncogene	circRNF13	Activate the Mek/Erk pathway and upregulate the expression of DNA repair proteins.	Shi et al. (2023a)
WTAP	Writer	BC	Oncogene	Bcl-2	Enhance the repair ability of double stranded DNA and protect cancer cells from apoptosis	Wang et al. (2023b)
YTHDC2	Reader	NPC	Oncogene	IGF1R	Accelerate the repair process of IR-induced DNA double-strand breaks, inhibition of IR-induced autophagy, and maintenance of the stemness of cancer cells.	Wang et al. (2023a)
HNRNPC	Reader	PC	Oncogene	RhoA	Trigger DNA repair and play an important role in regulating cell division.	Espinha et al. (2016)
ALKBH5	Eraser	HCC	Suppressor	/	Activate ERK/JNK signaling, protect cells from DNA damage and apoptosis, and rapidly induce G2/M arrest during stress	Xu et al. (2022b)
ALKBH5 YTHDF2	Eraser reader	CRC	Suppressor	CircAFF2	Enhance the radiation-induced DNA damage response, and lead to G2/M phase arrest	Zeng et al. (2022)

BC: breast cancer; CC: cervical cancer; CRC: colorectal cancer; GBM: glioblastoma; GSCs: glioma stem cells; HCC: hepatocellular carcinoma; HR: homologous recombination; IR: ionizing radiation; NHEJ: non-homologous end-joining; NPC: nasopharyngeal carcinoma; NSCLC: non-small cell lung cancer; PC: pancreatic cancer

The m6A “writers” are proteins that are capable of adding m6A modification sites. It consists of a methyltransferase complex, with METTL3 as its key catalytic component (Liu et al. 2014). Glioblastoma (GBM) is a malignancy with poor radiosensitivity, in which glioma stem cells (GSCs) are essential contributors to radioresistance owing to their ability to preferentially activate DNA damage checkpoints and promote HR (Bao et al. 2006). It has been shown that GBM tissues showed higher expression of METTL3 compared with normal brain tissues (Li et al. 2019). METTL3 enhances the efficiency of DNA damage repair by promoting the methylation of sex-determining region Y-box 2 (SOX2) and triggering the activation of the transcription of certain DNA repair genes, which contributes to the radioresistance of GSCs (Xie et al. 2022). Moreover, HR repair is crucial in the DNA damage repair pathway induced by the METTL3-SOX2 axis (Visvanathan et al. 2018). Wnt/β-catenin represents an evolutionarily conserved signaling pathway that targets the proto-oncogene c-Myc, triggers the activation of DNA damage checkpoints Chk1/2 and Ataxia telangiectasia mutated, and increases the production of HR- and NHEJ-related proteins, allowing for the repair of damaged DNA (Yang et al. 2021a). Previous studies have proven that METTL3 activates Wnt/β-catenin signaling and enhances DNA damage repair by mediating the m6A modification of LINC00839, to increase the stemness maintenance and radioresistance of GSCs (Yin et al. 2023). LINC00839 is classified as a long-stranded ncRNA that acts as a modular scaffold for conjugation to catenin (Huang et al. 2021), which in turn promotes c-Src-mediated β-catenin phosphorylation and activation of the Wnt/β-catenin signaling cascade. These studies indicate that METTL3 facilitates the expression of key proteins for DNA repair and thus, improves the survival of irradiated cells by mediating the m6A modification of SOX2 mRNA and LINC00839, which are major contributors to the radioresistance of GBM. Further exploration of m6A modifications in GBM may offer novel approaches for rescuing radioresistance.

Moreover, METTL3 and its associated m6A modifications were identified at higher levels in non-small cell lung cancer (NSCLC) cells than in adjacent normal tissues. They were strongly correlated with cell proliferation, migration, and invasion (Liu et al. 2020). In NSCLC cells exposed to carbon ion radiotherapy, METTL3 induces m6A modification of H2A histone family member X mRNA and suppresses its decay, causing both increased expression and DNA damage repair, ultimately contributing to NSCLC cell survival and radioresistance (Xu et al. 2023).

CXC motif chemokine ligand 1 (CXCL1) is a small-molecule cytokine whose secretion contributes to accelerated DNA damage repair through the activation of the MEK/ERK signaling pathway and ultimately enhances tumor radioresistance (Tsai et al. 2023). CircRNF13 is a novel circRNA regulated by m6A modifications that specifically down-regulates the expression of CXCL1 in cervical cancer (Shi et al. 2023a). METTL3 promotes m6A methylation and degradation of circRNF13, which leads to increased expression of CXCL1 and enhances radioresistance in cervical cancer (Shi et al. 2023a). Another study of esophageal squamous cell carcinoma confirmed this finding (Zhang et al. 2017). In summary, METTL3 and METTL3- mediated m6A modifications have been shown to enhance radioresistance by increasing the expression of DNA repair proteins in GBM and cervical and esophageal cancers. Nevertheless, the utility of METTL3 in other tumors still requires further investigation.

MELLT14 also serves as the core subunit of m6A methyltransferase, which possesses the ability to identify and directly bind histone 3 lysine 36 trimethylation (H3K36me3) without relying on the involvement of RNA polymerase II. This promotes the binding of the m6A methyltransferase complex to the neighboring RNA pol II, which then delivers m6A methyltransferase complex to new RNAs during transcriptome elongation, thereby increasing transcriptome-wide m6A abundance. Therefore, METTL14 is critical for binding the m6A methyltransferase complex to H3K36me3 (Zhou et al. 2021). Nevertheless, whether MELLT14 mediates m6A modifications to regulate tumor radiosensitivity remains to be explored.

RNA-binding proteins, colloquially referred to as "readers", possess the capability to interact with the m6A motif to assign specific phenotypic outcomes. YTHDF2 was found to stabilize MYC, VEGFA, and LINC00839 mRNAs within GBM through m6A-dependent mechanism (Dixit et al. 2021; Xu et al. 2020). The activation of Chk1/2 by MYC and the activation of Wnt/β-catenin pathways by LINC00839 can promote DNA damage repair, as well as VEGFA exhibits strong ability to promote CSC generation and vasculogenic mimicry formation, which are closely related to the lower radiation sensitivity of tumors. Bioinformatics studies have revealed that YTHDC2 is overexpressed in radioresistant nasopharyngeal carcinoma cells owing to the hypomethylation of its promoter (He et al. 2020). YTHDC2 engages in a physical interaction with insulin-like growth factor 1 receptor mRNA and upregulates its translation efficiency, which stimulates the phosphorylation of protein kinase B (also known as AKT) and ribosomal protein S6. The phosphorylation and subsequent activation of AKT play essential roles in the radioresistance of nasopharyngeal carcinoma (Tulalamba and Janvilisri 2012). AKT, a serine/threonine kinase, is the central factor in PI3K mediated radioresistance both in vivo and in vitro, and the PI3K/AKT signaling pathway has been shown to facilitate the repair of IR-induced DSBs (Florczak et al. 2009), the inhibition of autophagy, and the maintenance of the stemness of cancer cells (Chen et al. 2023). AKT also interacts with DNA-dependent protein kinase catalytic subunits to form a functional complex that promotes the accumulation and phosphorylation of DNA-dependent protein kinase catalytic subunits at DNA damage sites, which in turn activates the NHEJ pathway (Yang et al. 2021b) and significantly increases tumor radioresistance (Guo et al. 2021; Ma et al. 2021).

The eraser recognizes methylation sites of adenine and cytosine in single-stranded DNA and RNA (Trewick et al. 2002), and removes m6A modifications, mainly Fat mass and obesity-associated protein (FTO) and α-ketoglutarate-dependent dioxygenase AlkB homologue 5 (ALKBH5) (Yu et al. 2019). ALKBH5 is phosphorylated and SUMOylated via the ROS-activated ERK/JNK signaling pathway, which reduces substrate accessibility and blocks mRNA m6A demethylase activity, leading to increased mRNA m6A methylation (Lin et al. 2023). Furthermore, there is a positive feedback loop in which ROS selectively promotes the transcription of METTL3 and METTL14 through ALKBH5 SUMOylation, further enhancing m6A levels (Xu et al. 2022b). Previous studies have shown that radiation-induced ROS protects cells from DNA damage and apoptosis by regulating mRNA m6A levels via ALKBH5 and rapidly inducing G2/M blockade, which in turn enhances the radioresistance of hepatocellular carcinoma (Yu et al. 2021).

SETD2 is a methyltransferase that specifically catalyzes the trimethylation of histone H3 at lysine 36 (Niu et al. 2020). Previous studies have suggested that SETD2 inhibits lung adenocarcinoma cell apoptosis and reduces radiosensitivity in vitro by regulating intracellular HR, NHEJ, and MMR repair mechanisms via H3K36me3 (Shi et al. 2023b). In a recent study, SETD2 presented a significant regulatory relationship with m6A modification and maintained chromatin integrity by contributing to DNA repair (Zeng et al. 2022). Another study discovered that the absence of SETD2 downregulates the level of m6A in RNA in gliomas and exerts a crucial function in suppressing tumorigenesis. The above studies indicate that the regulation of tumor radiosensitivity by STED2 may be related to m6A modification; however, the specific mechanism remains unknown and further studies are required to confirm this.

CircAFF2 is a new circular RNA that has been demonstrated to bind CAND1, stimulate the binding of CAND1 and Cullin1, and inhibit its analog modifications, thereby enhancing the radiation-induced DNA damage response and leading to G2/M phase arrest. This process increasing the radiosensitivity and improving the prognosis of patients with colorectal cancer (Shao et al. 2023). Modulation of radiosensitivity by CircAFF2 is closely correlated with m6A modifications, with a focus on ALKBH5-mediated demethylation and YTHDF2-regulated degradation of circAFF2.

**Fig. 2:**
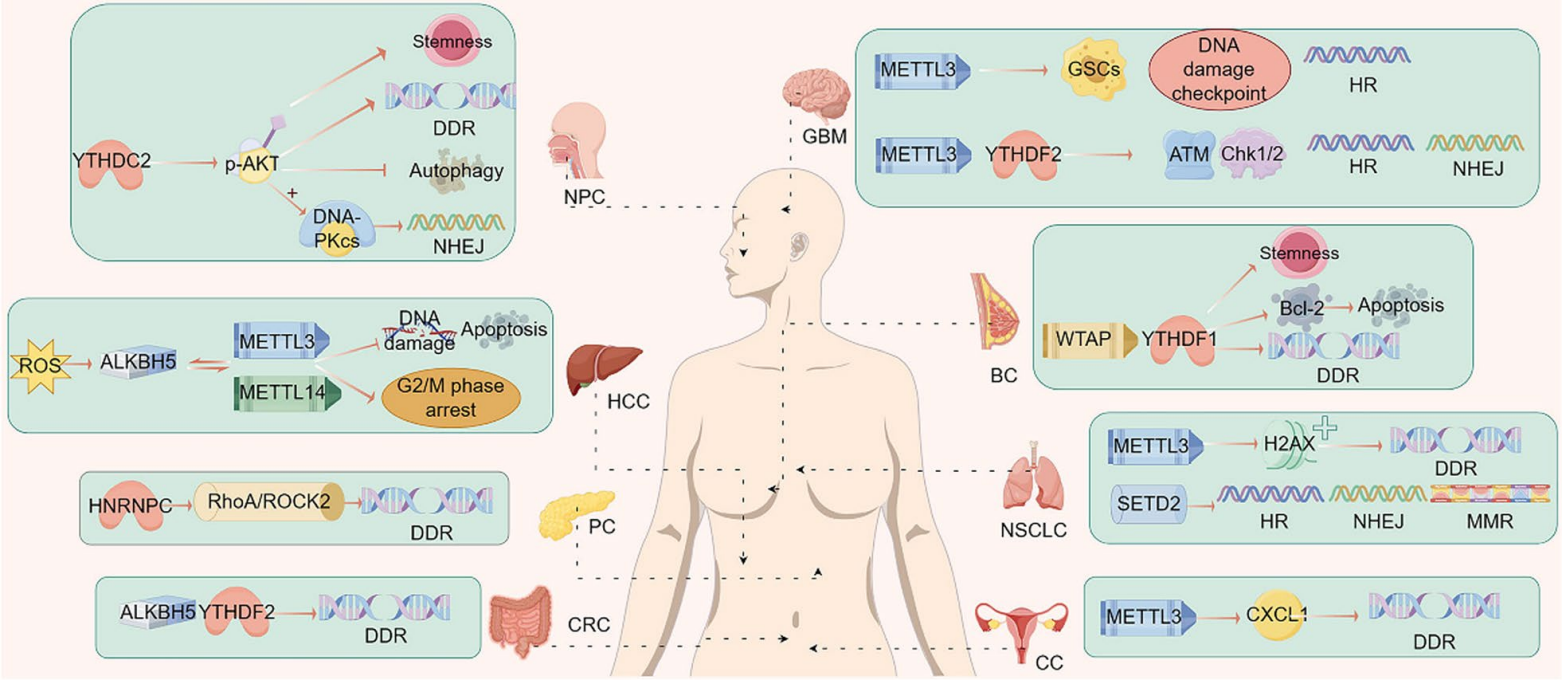
The mechanism by which m6A regulatory factors affect radiosensitivity in various tumors (By Figdraw). The m6A methylation process can affect radiosensitivity by activating certain DNA damage and repair pathways. BC: breast cancer; CC: cervical cancer; CRC: colorectal cancer; DDR: DNA damage repair; GBM: glioblastoma; GSCs: glioma stem cells; HCC: hepatocellular carcinoma; HR: homologous recombination; MMR: mismatch repair; NHEJ: non-homologous end-joining; NPC: nasopharyngeal carcinoma; NSCLC: non-small cell lung cancer; PC: pancreatic cancer; ROS: reactive oxygen species

Neuropilin-1 (NRP1) is a type I transmembrane glycoprotein that primarily acts as a coreceptor by complexing with other transmembrane receptors (Douyère et al. 2022). Ionizing radiation promotes NRP1 expression (Shao et al. 2021), and NRP1 upregulation enhances stem cell properties, leading to resistance to radiotherapy. As a member of the methyltransferase complex, WTAP lacks methyltransferase activity, but can induce the recruitment and localization of METTL3/14 by acting as a binding protein. In a breast cancer study, NRP1 overexpression was found to interfere with the m6A-dependent downregulation of the Bcl-2 mRNA via WTAP and improve DNA repair ability, which in turn protects breast cancer cells from apoptosis and promotes radioresistance. Additionally, the m6A reader YTHDF1 positively regulates the translation of Bcl-2 protein in breast cancer cells by recognizing and binding to m6A sites (Wang et al. 2023b). The mechanism affecting radiosensitivity warrants further investigation (Fig. 2).

### Regulation of cell cycle arrest by m6A modifications

The cell cycle comprises a series of sequential events that ensure cell growth and division (Pack et al. 2019). Cell cycle arrest is triggered by IR-induced DNA damage, and allows sufficient time for DNA damage repair before the cell re-enters its normal cycle. IR-induced cell cycle arrest is mainly regulated by three main cell cycle checkpoints: G1/S-phase arrest, S-phase arrest, and G2/M-phase arrest. Among these, the G2/M blockade is correlated with tumor radiosensitization. Checkpoints delay cell cycle progression in response to stimuli or induce cell cycle withdrawal or cell death when DNA damage is irreparable, which enforces the orderly continuation of the cell cycle and prevents DNA over-replication (Matthews et al. 2022).

Polo-like kinases (PLKs) are a family of serine/threonine protein kinases that are widely expressed in eukaryotic cells (Wang et al. 2021). However, they are more highly expressed in the majority of cancers, and are associated with poor prognosis in patients with cancer. PLK1 exerts its main effects by regulating the initiation and completion of cell division, mitosis, the DNA damage response, and the maintenance of genome stability. Abnormal PLK1 expression triggers cell cycle checkpoints that drive tumorigenesis and chromosomal instability (Liu et al. 2017). PLK1 inhibition induces G2/M phase arrest and increases DSBs. It has been demonstrated that METTL3 expression is higher in pancreatic adenocarcinoma tissues than in healthy pancreatic tissues. METTL3 upregulates PLK1 expression by methylating the PLK1 3'UTR and contributes to the radioresistance of pancreatic cancers in a cell cycle-dependent manner (Tatekawa et al. 2022). Furthermore, IGF2BP2, a protein bound to methylated RNA, prevents the decay of m6A-modified mRNAs and promotes RNA stabilization and translation. IGF2BP2 also binds to the m6A site of the PLK1 3'UTR in pancreatic cancer cells, upregulates the expression of PLK1, thereby maintaining cell cycle homeostasis and regulateing radiosensitivity (Chen et al. 2021). Demethylation of this site causes replication stress via dissociation of IGF2BP2 because PLK1 is downregulated in the G2/M phase instead of upregulated. The outcomes are mitotic catastrophe and increased radiosensitivity.

**Fig. 3:**
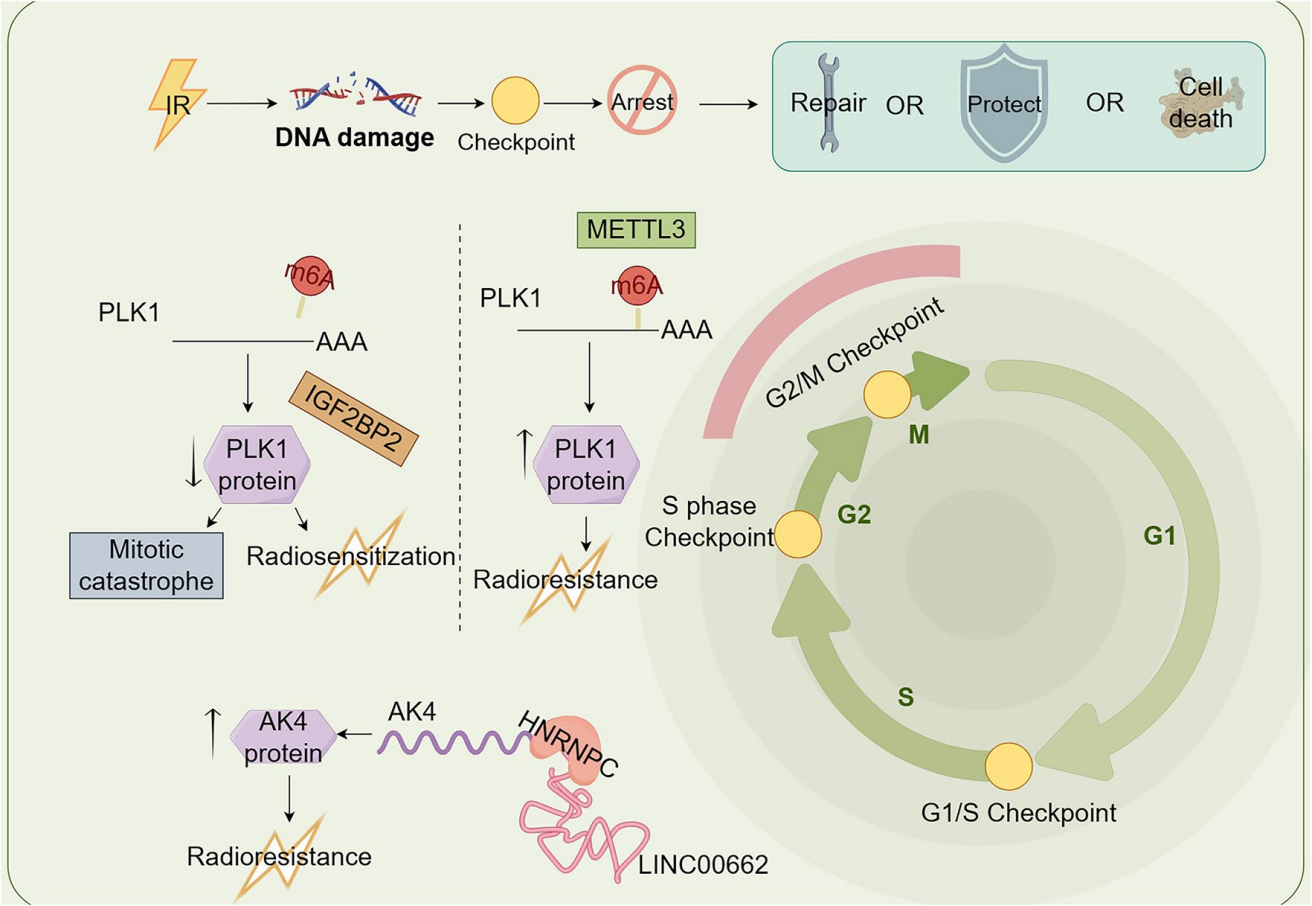
The occurrence of DNA damage caused by ionizing radiation can activate cell cycle checkpoints to regulate cell cycle arrest, providing time for repairing and avoiding radiation damage before the cells re-enter the normal cycle, in order to enhance radioresistance (By Figdraw). The expression of PLK1 and AK4 regulates radiation sensitivity by interfering with cell cycle arrest. IR: ionizing radiation

A recent study revealed that LINC00662 is overexpressed in oral squamous cell carcinoma (OSCC) compared with adjacent noncancer tissues and serves as a new oncogenic lncRNA (He et al. 2021). In OSCC, the m6A reader HNRNPC is identified a survival-related splicing factor that interacts with LINC00662 or adenylate kinase 4 (AK4). Gain-of-function assays have shown that overexpression of AK4 inhibits OSCC cell apoptosis and cell cycle arrest and promotes resistance to radiation (Chen et al. 2020). LINC00662 promotes AK4 mRNA stability by recruiting the HNRNPC protein, thus exerting pro-radioresistance effects in OSCC cells. However, whether cell cycle arrest participates in the regulation of radiosensitivity through the interaction of HNRNPC with AK4 remains unclear and requires further exploration (Fig. 3).

### Regulation of cancer stem cell generation by m6A modification

Cancer stem cells (CSCs), also known as tumor-initiating cells, exhibit high resistance, leading to failure of antitumor therapy, tumor recurrence, and metastasis (Zhong et al. 2019). CSCs exhibit radioresistance due to their strong capacity to repair DNA, (Zhu et al. 2017). scavenge ROS, and self-renew.

MiR-99a-5p is a tumor suppressor, whose high expression has been confirmed to be correlated with the suppression of CSCs and radiosensitization. METTL14 upregulates miR-99a-5p by positively regulating m6A-mediated DGCR8-dependent processing of pri-mir-99a, which in turn enhances the radiosensitivity of esophageal squamous carcinoma (Liu et al. 2021).

**Fig. 4:**
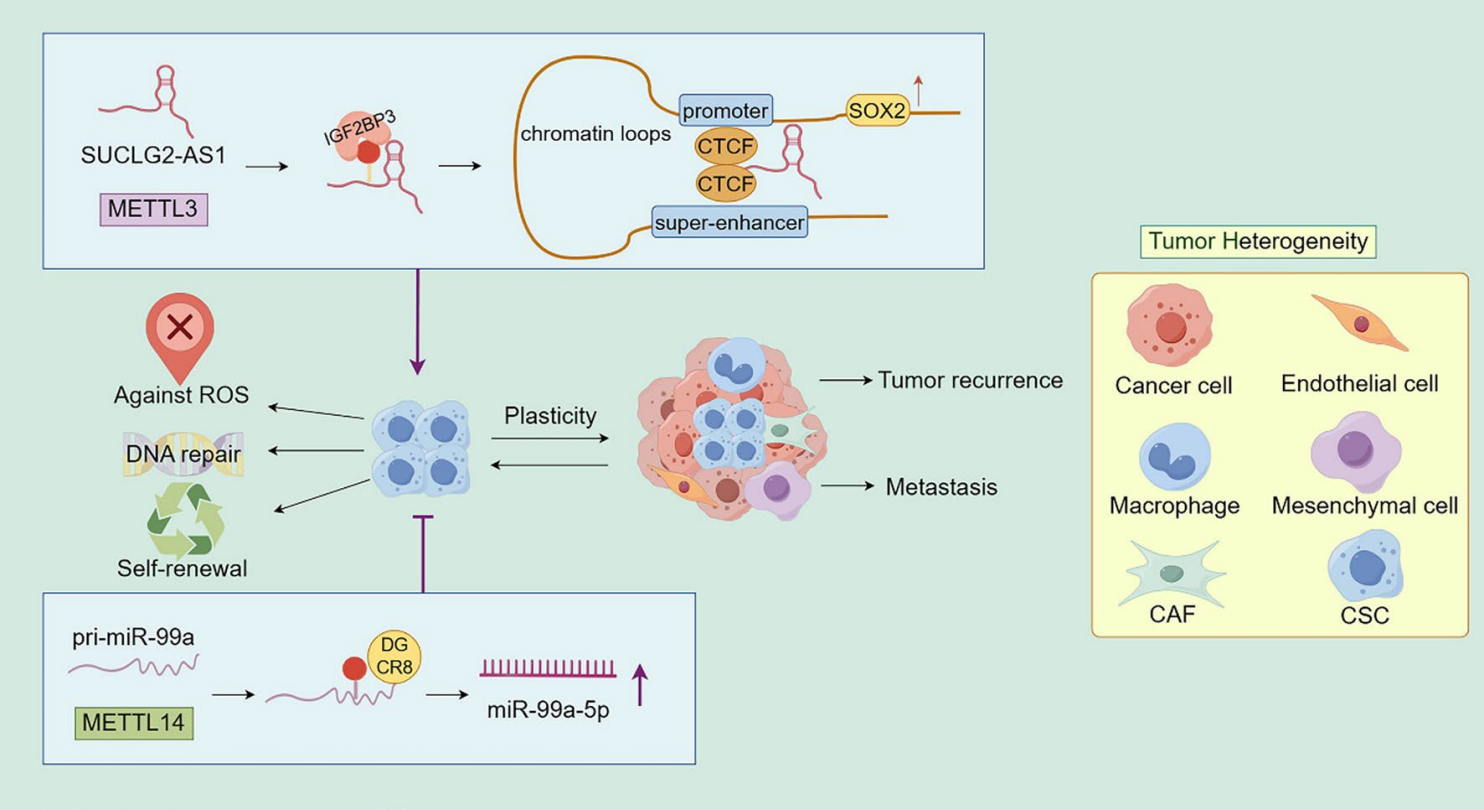
CSCs enhance the radioresistance of tumors by altering their structure, endowing them with powerful DNA repair ability, defense ability against reactive oxygen species, and self-renewal ability, ultimately leading to tumor recurrence and metastasis (By Figdraw). m6A modification regulates cell stemness by regulating miR-99a-5p and SUCLG2-AS1, thereby affecting radiosensitivity. CAF: cancer associated fibroblast; CSC: cancer stem cells; ROS: reactive oxygen species

The super‐lncRNA SUCLG2‐AS1 was upregulated in nasopharyngeal carcinoma tissues, and inhibited apoptosis and radiosensitivity. SUCLG2-AS1 enhances the transcription and induces the accumulation of SOX2 by mediating the interaction of CTCF (a multifunctional transcription factor) with the enhancer and promoter regions of SOX2. High expression of SOX2 is associated with maintenance of cellular stemness, and upregulation of β - catenin signaling (Wang et al. 2023c). SUCLG2 - AS1 is expressed in a m6A-dependent manner. First, SUCLG2-AS1 is expressed as m6A in the nucleus by METTL3; then, IGF2BP3 recognizes and reads SUCLG2-AS1 in the cytoplasm, which in turn prevents its degradation to maintain the stability of SUCLG2-AS1 and contributes to its entry into the nucleus. This positive mechanism upregulates SOX2 expression and radioresistance in nasopharyngeal carcinoma (Yu et al. 2023). The m6A modification of radiosensitivity by regulating the stemness characteristics of tumor stem cells promises to be a breakthrough in radiosensitization (Fig. 4).

### Regulation of the immune microenvironment and other pathways by m6A modifications

The TME includes all noncancerous cells in the tumor, such as blood and lymphatic vessels, stromal cells (fibroblasts), immune cells, the extracellular matrix (ECM), and soluble biochemical factors (Xiao and Yu 2021)

**Fig. 5:**
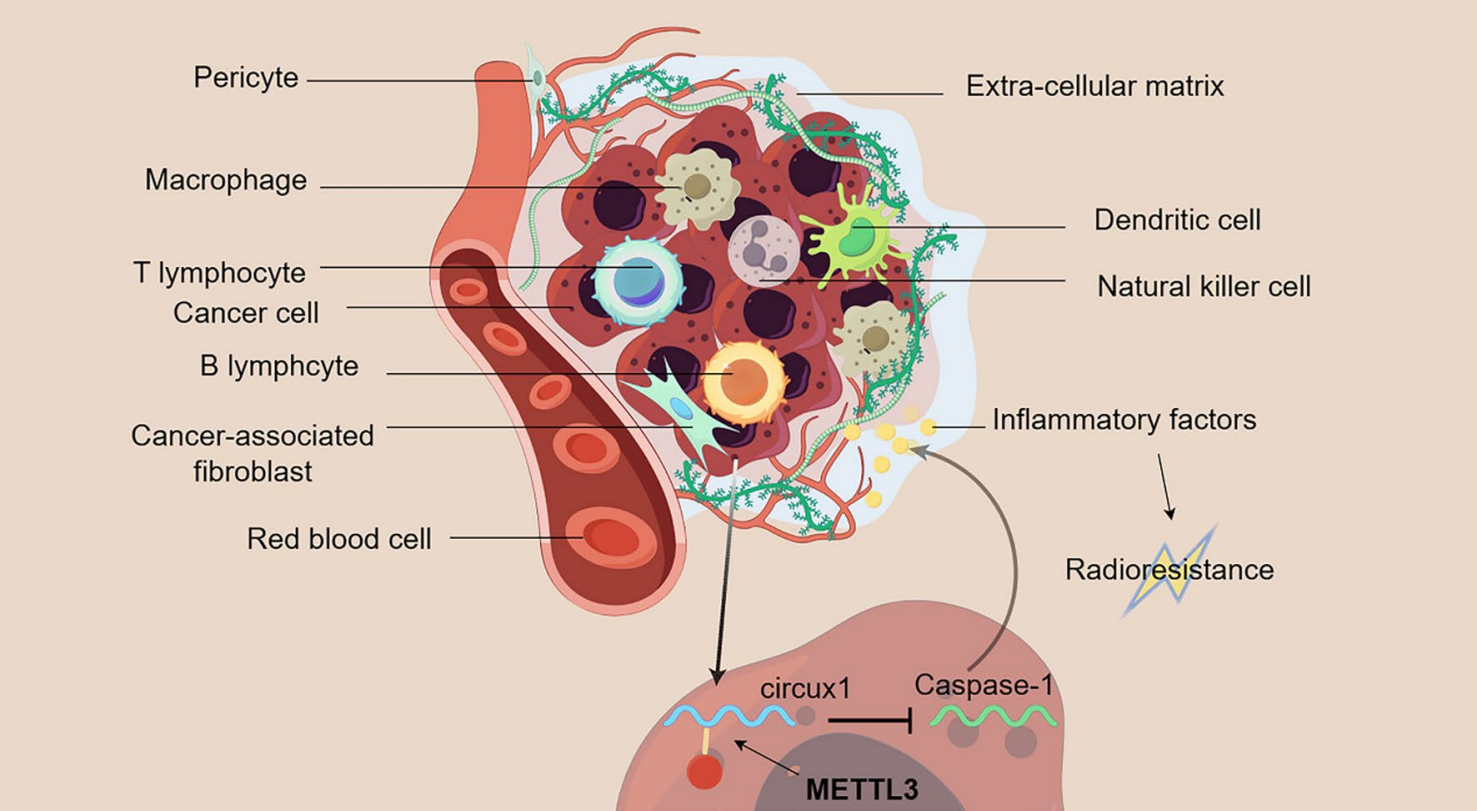
The tumor microenvironment refers to the surrounding microenvironment in which tumor cells exist, including surrounding blood vessels, immune cells, cancer-associated fibroblasts, various signaling molecules, and the extracellular matrix (ECM) (By Figdraw). m6A methylation inhibits the expression of caspase-1 by upregulating circux1, leading to the release of inflammatory factors in the tumor microenvironment and radioresistance of hypopharyngeal carcinoma

Caspase-1 is the first cysteinyl aspartate specific proteinase family member, also known as ice-interleukin-1β converting enzyme (Winter et al. 2004) Caspase-1 regulates the tumor inflammatory microenvironment and induces programmed cell death and radiosensitization in malignant tumors. It has been demonstrated that METTL3 mediates m6A methylation of circux1 to stabilize its expression in the cytoplasm. Circux1 inhibits the expression of caspase 1 mRNA, leading to the release of inflammatory factors and radioresistance in hypopharyngeal carcinoma (Fig. 5) (Wu et al. 2021).

Ferroptosis, a recently identified cell death process characterized by iron accumulation and lipid peroxidation up-regulation contributes to radiosensitisation. FTO acts as a m6A demethylase, eliminating the m6A modification of the OTUB1 (Ovarian Tumor Family Deubiquitinase) transcript and promoting the expression of OTUB1, which protects against radiation-induced ferroptosis (Koppula et al. 2021).

## Conclusion

In summary, tumor radioresistance emerges as a multifaceted process, intricately modulated by a diverse array of factors encompassing DNA damage repair, cell cycle arrest, cancer stem cell dynamics, hypoxic conditions, alterations in the TME, and autophagy. In recent years, m6A modification has garnered significant attention as a research focus in the post-transcriptional regulation of gene expression. However, the structures, functions, and regulatory mechanisms of radioresistance in malignancies have only recently been studied. In this review, we summarize the phenomena, mechanisms, and target genes of m6A modifications regulating radioresistance that have been discovered to date, aiming to provide new methods for radiosensitization.

## Data Availability

No datasets were generated or analysed during the current study.

## Abbreviations

AK4: Adenylate kinase 4
ALKBH5: AlkB homologue 5
BC: Breast cancer
Bcl-2: B-cell lymphoma-2
CAND1: Cullin-associated and neddylation-dissociated protein 1
CC: Cervical cancer
Chk1/2: Checkpoint kinases 1/2
CRC: Colorectal cancer
CSCs: Cancer stem cells
CTCF: CCCTC-binding factor
CXCL1: CXC motif chemokine ligand 1
DGCR8: DGCR8 microprocessor complex subunit
DSBs: DNA double-strand breaks
ECM: Extracellular matrix
ERK: Extracellular regulated protein kinases
FTO: Fat mass and obesity-associated protein
GBM: Glioblastoma
GSCs: Glioma stem cells
H2AX: H2A histone family member X
H3K36me3: Histone 3 lysine 36 trimethylation
HCC: Hepatocellular carcinoma
HNRNPC: Heterogeneous nuclear ribonucleoprotein C
HR: Homologous recombination
IGF2BP: Insulin-like growth factor 2 mRNA-binding protein
IR: Ionizing radiation
JNK: C-jun N-terminal kinase
LUAD: Lung adenocarcinoma
MEK: MAP kinase-ERK kinase
METTL3: Methyltransferase Like 3
METTL14: Methyltransferase Like 14
MMR: Mismatch Repair
MYC: Myelocytomatosis
ncRNA: Non-coding RNA
NHEJ: Non-homologous end joining
NPC: Nasopharyngeal carcinoma
NRP1: Neuropilin-1
NSCLC: Non-small cell lung cancer
OSCC: Oral squamous cell carcinoma
PC: Pancreatic cancer
PI3K: Phosphatidylinositide 3-kinases
PLKs: Polo like kinases
PKB: Protein kinase B
ROS: Reactive oxygen species
S6: Ribosomal protein S6
SETD2: SET domain-containing protein 2
SOX2: Sex-determining region Y-box 2
TME: Tumor microenvironment
VEGFA: Vascular endothelial growth factor A
WTAP: Wilms tumor 1 associated protein
YTHDC: YTH domain-containing
YTHDF: YTH domain-containing family protein
